# The impact of the Sepsis-3 definition on ICU admission of patients with infection

**DOI:** 10.1186/s13049-019-0680-9

**Published:** 2019-11-04

**Authors:** Jenny Klimpel, Lorenz Weidhase, Michael Bernhard, André Gries, Sirak Petros

**Affiliations:** 1 Medical ICU, University Hospital of Leipzig, Liebigstr. 20, 04103 Leipzig, Germany; 2 Emergency Department, University Hospital of Düsseldorf, Düsseldorf, Germany; 3 Emergency Department, University Hospital of Leipzig, Leipzig, Germany

**Keywords:** Sepsis, qSOFA, SOFA score, Intensive care, Infection, Mortality, Emergency department

## Abstract

**Background:**

Sepsis is defined as a life-threatening organ dysfunction due to a dysregulated inflammation following an infection. However, the impact of this definition on patient care is not fully clear. This study investigated the impact of the current definition on ICU admission of patients with infection.

**Methods:**

We performed a prospective observational study over twelve months on consecutive patients presented to our emergency department and admitted for infection. We analyzed the predictive values of the quick sequential organ failure assessment (qSOFA) score, the SOFA score and blood lactate regarding ICU admission.

**Results:**

We included 916 patients with the diagnosis of infection. Median age was 74 years (IQR 62–82 years), and 56.3% were males. There were 219 direct ICU admissions and 697 general ward admissions. A qSOFA score of ≥2 points had 52.9% sensitivity and 98.3% specificity regarding sepsis diagnosis. A qSOFA score of ≥2 points had 87.2% specificity but only 39.9% sensitivity to predict ICU admission. A SOFA score of ≥2 points had 97.4% sensitivity, but only 17.1% specificity to predict ICU admission, while a SOFA score of ≥4 points predicted ICU admission with 82.6% sensitivity and 71.7% specificity. The area under the receiver operating curve regarding ICU admission was 0.81 (95 CI, 0.77–0.86) for SOFA score, 0.55 (95% CI, 0.48–0.61) for blood lactate, and only 0.34 (95% CI, 0.28–0.40) for qSOFA on emergency department presentation.

**Conclusions:**

While a positive qSOFA score had a high specificity regarding ICU admission, the low sensitivity of the score among septic patients as well as among ICU admissions considerably limited its value in routine patient management. The SOFA score was the better predictor of ICU admission, while the predictive value of blood lactate was equivocal.

## Background

Sepsis is a global medical challenge with a very high incidence and mortality [1–3]. The formulation of a meaningful definition of sepsis remains a challenge, balancing between a clinically prudent definition and that appropriate for research purposes. While the first and second consensus definitions of sepsis were based on the presence of systemic inflammation [4, 5], sepsis is currently defined as a life-threatening organ dysfunction due to a dysregulated host response to infection [6–8]. The Sequential Organ Function Assessment (SOFA) Score, which has been in use in intensive care units (ICU) for more than two decades [9], is now at the center of the definition of sepsis, and this score is found to be a better prognostic indicator [6].

It is essential to diagnose sepsis at an early stage to positively influence the clinical outcome by initiating appropriate management bundles [10]. The introduction of the quick SOFA (qSOFA) score thus represents the effort to identify high risk patients as early as possible by using basic clinical criteria instead of complex biomarkers [8]. The score includes respiratory rate, Glasgow Coma Scale and systolic blood pressure, based on the analysis preceding the current sepsis definition algorithm. A score of at least two points is considered positive. Several publications have reported the correlation between a positive qSOFA and poor outcome in sepsis patients [6, 11–15]. However, although the qSOFA score represents the shift from the previous notion of systemic inflammation now to organ dysfunction as the mainstay of sepsis definition, its impact on the decision-making process is controversial.

Our primary aim was to investigate how the current sepsis criteria influence the decision in the emergency department on the inpatient level of care of patients with infection. Our secondary aim was to assess the impact of the current sepsis criteria on clinical outcome.

## Methods

We conducted a prospective non-interventional observational study at the University Hospital of Leipzig from 01 May 2017 to 30 April 2018. The study was approved by the ethics commission of the University of Leipzig and conducted according to the ethical principles outlined in the Helsinki Accord. For the sake of homogeneity, we have only screened patients with non-surgical infections. Inclusion criterion was the diagnosis of infection based on clinical, laboratory, radiological and/or microbiological evidence, in accordance with published international consensus guidelines for the diagnosis of infections [16–20]. Exclusion criteria were surgical procedure considered a possibility during ward admission, pregnancy, age < 18 years, refusal to participate in the study, readmission during the study period, and decision for end-of-life care on admission to the emergency department (ED).

We recorded during the ED management demographic data, the presumed or certain focus of infection, the qSOFA score and blood lactate for every patient with presumed infection admitted to either the medical ICU or the general medical wards. Sepsis was defined based on the current Sepsis-3 recommendations as an infection plus at least two new SOFA score points. The SOFA score was computed for patients with the clinical suspicion of sepsis in accordance with the recommendations of the current sepsis definition algorithm [8]. In short, the SOFA score was calculated if in a patient with infection the qSOFA score was positive or, in case qSOFA was negative, if the ED physicians considered sepsis likely. The ED physicians were trained and experienced in applying the algorithms in the management of sepsis. Regarding the SOFA score, the score points presented in this study are not the total score points, but rather the score points considered associated with the current infection. For this purpose, previous electronic patient charts of the hospital as well as information supplied by patients or their guardians or family physicians were evaluated looking for chronic conditions that would influence the SOFA score. In case of missing information, the study group judged the clinical validity of the present variables in relation to the current infection and the SOFA score calculated accordingly.

Additionally, we recorded data on chronic underlying diseases (neurological illnesses requiring home support, chronic pulmonary disease requiring home oxygen therapy or non-invasive ventilation or that resulted in significant limitations in quality of life, chronic heart failure New York Heart Association grade III or IV, liver cirrhosis Child-Pugh class B or C, end-stage renal failure, active hematological malignancy, active solid malignancy and current immunosuppressive therapy).

The medical ICU applies an integration model of intensive care and intermediate care, which allows a flexible use of staff and logistic capacity. The decision for ICU admission was based on published recommendations for intensive care and intermediate care patients [21, 22]. Thus, the ICU cohort in this study includes both patient subgroups managed by the same team of intensivists and ICU nurses. We analyzed the total ICU cohort as a group, because we believed that further stratification into ICU and intermediate care group would not add any relevant information regarding the main aim of the study. For patients admitted to the ICU, we additionally collected the following data: Acute Physiology And Chronic Health Evaluation (APACHE) II score, the need for vasopressor therapy, invasive mechanical ventilation and renal replacement therapy (RRT), and ICU mortality. We also recorded hospital mortality for the total cohort.

Data were collected daily on an Excel sheet and then transferred to the SPSS sheet after validation by the study group. We performed the statistical analysis using SPSS for Windows version 24 (IBM SPSS, USA). Numerical data are given as either mean with standard deviation or median with interquartile range (IQR) depending on whether they are normally distributed based on Shapiro-Wilk and Kolmogorov-Smirnov tests. We compared numerical data using either the Student t test or the Mann-Whitney U test depending on their normal distribution. Categorical variables were tested using the chi square test with two-sided significance. We calculated the sensitivity, specificity, positive and negative predictive values of the following variables to predict ICU admission: a qSOFA of ≥2 points, a blood lactate of > 2 mmol/l, and a SOFA score of ≥2 and ≥ 4 points. We constructed receiver operating characteristics (ROC) curves and calculated the area under the ROC (AUROC) regarding ICU admission for positive qSOFA (≥2 points) as well as for the SOFA score and blood lactate as continuous variables. A *p* value <0.05 was considered statistically significant.

## Results

We included 916 patients with a median age of 74.0 years (IQR 62–82 years), of whom 56.3% were males. There were 219 (23.9%) patients directly admitted to the ICU, of whom 166 (75.8%) fulfilled ICU admission criteria and 53 (24.2%) fulfilled intermediate care criteria. The other 697 (76.1%) patients were admitted to the general wards. Baseline characteristics of the study population are presented in Table 1. Six patients in the ICU admission group and 10 patients in the general ward group with a positive qSOFA score did not fulfill the criteria for sepsis. Chronic lung diseases (15.1% versus 5.5%, *p* < 0.0001) and chronic heart failure (19.7% versus 9.6%, p < 0.0001) were significantly more common among ICU admissions than among general ward admissions, while solid malignancies were significantly more common among general ward admissions than among ICU admissions (16.5% versus 8.7%, *p* = 0.004).

**Table 1:** Baseline characteristics of the study population

Variable	Total cohort	ICU admission	General ward admission	p
N (% total)	916	219 (23.9)	697 (76.1)	
Male (%)	56.3	58.4	55.7	0.48
Median Age (years) with IQR	74.0 (62.0–82.0)	70.0 (59.0–78.0)	75.0 (62.0–83.0)	0.013
qSOFA ≥2 points (%)	23.6	39.9	12.8	<0.0001
≥1 chronic disease (%)	60.7	64.4	59.5	0.21
Focus of infection (%)				0.001
Respiratory	56.8	71.7	52.1	
Urogenital	24.6	14.6	27.7	
Abdominal	7.5	4.6	8.5	
Soft tissue	5.6	3.7	6.2	
Others	5.5	5.6	5.6	

*IQR* interquartile range

**Fig. 1:**
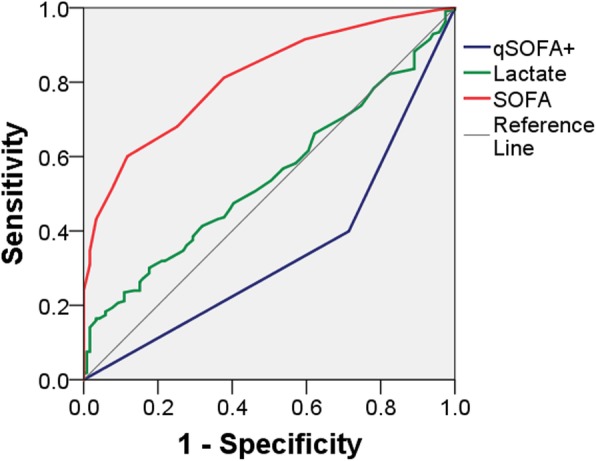
Receiver operating characteristic curves of the SOFA score, blood lactate and a positive qSOFA for ICU admission based on emergency department admission data. The AUROC was 0.82 (95% CI, 0.77–0.86) for SOFA score, 0.55 (95% CI, 0.48–0.61) for blood lactate level, and 0.34 (95% CI, 0.28–0.40) for a positive qSOFA

The sensitivity, specificity, positive and negative predictive values of qSOFA, blood lactate and SOFA score in the ED regarding ICU admission are presented in Table 2. The ROC curves of the SOFA score, blood lactate and a positive qSOFA for ICU admission are shown in Fig. 1. The highest AUROC regarding the prediction of ICU admission was demonstrated for SOFA score, while the figure for blood lactate was equivocal and that for qSOFA was low.

**Table 2:** Sensitivity, specificity, positive and negative predictive values of qSOFA, blood lactate and the SOFA score regarding ICU admission

Variable	Sensitivity (%)	Specificity (%)	Positive predictive value (%)	Negative predictive value (%)
qSOFA ≥2 points	39.9	87.2	49.1	82.5
Blood lactate > 2 mmol/l	58.9	58.1	31.5	81.3
SOFA ≥2 points	97.3	17.1	67.6	77.8
SOFA ≥4 points	81.3	71.7	79.1	65.0

Eighteen patients out of the general ward group (2.6%) had to be transferred to the ICU within 48 h of admission due to clinical deterioration. Only 5/18 (27.8%) had a positive qSOFA score on ED admission.

The median SOFA score for patients with sepsis on ED admission was 7.0 (4.0–10.5) for the ICU admission group and 3.0 (2.0–5.0) for the general ward group (*p* < 0.001).

Data on disease severity in the ICU for the direct ICU admissions are given in Table 3.

**Table 3:** Disease severity for the ICU admission group during their ICU stay

Variable	
Mean APACHE-II score	24.7 ± 7.0
Mean SOFA score	7.4 ± 4.0
SOFA score ≥ 2 points (%)	97.3
Median Blood lactate (mmol/l) (with IQR)	1.6 (1.1–2.6)
Blood lactate > 2.0 mmol/l (%)	33.3
Invasive ventilation (%)	45.7
Vasopressor use (%)	53.9
RRT (excluding patients with ESRD) (%)	20.1
ICU mortality (%)	23.7
Hospital mortality (%)	30.0

*ESRD* end-stage renal disease, *IQR* interquartile range, *RRT* renal replacement therapy

A qSOFA score of ≥2 points had 52.9% sensitivity and 98.3% specificity regarding sepsis diagnosis. The hospital mortality rate was 44.7% among qSOFA-positive ICU admissions, but only 20.3% among qSOFA-negative ICU admissions (*p* < 0.0001). The hospital mortality in the general ward group was 13.9% for qSOFA-positive patients, but only 3.7% for qSOFA-negative patients (*p* = 0.001). The hospital mortality rate was 38.8% among lactate-positive (blood lactate > 2.0 mmol/l) ICU admissions, but only 17.8% among lactate-negative (blood lactate ≤2.0 mmol/l) ICU admissions (*p* = 0.001). The hospital mortality in the general ward group was 13.2% for lactate-positive patients, but only 4.1% for lactate-negative patients (*p* < 0.0001).

## Discussion

This observational study prospectively evaluated the predictive value of the current sepsis definition on ICU admission of patients with infection presenting to the emergency department. The study showed that the SOFA score had a strong correlation with ICU admission. A mere positive SOFA score, defined as at least two score points, used for sepsis definition showed a high sensitivity, but a very low specificity to predict ICU admission in our cohort. A SOFA score of at least four points showed a good sensitivity and specificity to predict ICU admission. This cut-off may help in management decisions in the ED regarding the level of inpatient care. However, this should be independently validated.

In our study, only approximately 40% of patients directly admitted to the ICU fulfilled the qSOFA criteria. Moskowitz et al. have reported that qSOFA had a high specificity but a poor sensitivity to predict critical care interventions such as vasopressor therapy, assisted ventilation, intravenous fluid substitution, placement of invasive catheters or renal replacement therapy within the first 48 hours [23]. A retrospective study using electronic health records of patients treated for sepsis in the ED reported that qSOFA performed poorly for identifying sepsis in the ED and relying on qSOFA alone may delay initiation of interventions [24]. In a retrospective analysis of data from more than 30,000 unselected patients in the ED, the sensitivity of qSOFA regarding ICU admission or death was only 53.6% [25]. The decision to admit septic patients to high level care unit is usually dependent on particular organ dysfunction states. A prospective observational study concluded that clinical judgement is a fast and reliable method to stratify between ICU and general ward admissions in ED patients with sepsis, with qSOFA not adding value to this stratification [26].

The main limitation of qSOFA regarding management decision is its low sensitivity. Szkarmany et al. reported that the risk of missing patients with organ dysfunction while using qSOFA was 30.5% [27]. Williams et al. have also reported that the poor sensitivity of qSOFA may limit its utility as a bedside screening tool [28]. A meta-analysis showed that the initial qSOFA score is of limited prognostic value in ED patients with infections [29]. A prospective study in patients admitted with infection to the ED reported that qSOFA failed to identify two-thirds of patients with severe sepsis [30]. Another retrospective analysis of data including 130,595 adult visits to the ED also concluded that qSOFA is a poor tool for ED sepsis screening [31].

Our study confirmed that patients with a positive qSOFA have a significantly higher hospital mortality rate than those with a negative qSOFA [11, 27, 28, 32–34]. Franchini et al. concluded in their meta-analysis that qSOFA is a higher rule-in than rule-out tool to predict mortality [35].

Our study has certain limitations. Firstly, it was monocentric and non-interventional. However, our data represent the clinical reality in the management of patients with sepsis. Secondly, we have included only non-surgical patients, so that one should be cautious in extrapolating our results to other patient populations. Thirdly, although we have taken every effort to diagnose infection based on available guidelines, we cannot rule out that our cohort might have included patients without any infection. The diagnosis of infection in the first hours of patient presentation remains a challenge. Fourthly, computing the qSOFA score might particularly be challenging in the aging population with a high prevalence of chronic underlying diseases, such as in our cohort. Therefore, these issues should be taken into consideration while comparing our results with those from other studies. Furthermore, we have computed the SOFA score only in those patients with a positive qSOFA and, in case of a negative qSOFA, in those patients with a high clinical suspicion of sepsis, which is the recommended approach by the current definition algorithm [8]. Such a clinical suspicion may be variable depending on the training and experience of emergency physicians. Although we have established the definition algorithm in our ED, we cannot rule out that we may have overlooked patients with sepsis. However, the low hospital mortality among our patients managed in the general wards may support our assumption that we could not have overlooked any significant number of sepsis patients. There is no evidence that computing the SOFA score in every admitted patient with infection is meaningful. Calculating the SOFA score may take time while waiting for laboratory results. However, we did not look into this issue and it is also not clear whether this delay might have a significant influence on patient management.

## Conclusion

The SOFA score has a strong impact on ICU admission of patients with infection. An infection-associated SOFA score of at least four points may help in decision making regarding high-level patient care. However, this cut-off should be prospectively validated. Although a positive qSOFA score has a high specificity to diagnosis sepsis and it correlates well with poor outcome, its low sensitivity makes its implementation in the decision-making process questionable.

## Data Availability

The datasets during the current study are available from the corresponding author on reasonable request.
